# Diagnostic potential of cell-free fetal nucleic acids in predicting pregnancy complications: A systematic review and meta-analysis on trisomy, pre-eclampsia, and gestational diabetes

**DOI:** 10.18502/ijrm.v23i2.18476

**Published:** 2025-05-01

**Authors:** Jiut Ram Keshari, Pritam Prakash, Seema Rani Sinha, Prem Prakash, Kirti Rani, Tarique Aziz, Shaily Shilpa

**Affiliations:** ^1^Department of Biochemistry, Indira Gandhi Institute of Medical Sciences, Patna, Bihar, India.; ^2^Department of General Surgery, Indira Gandhi Institute of Medical Sciences, Patna, Bihar, India.; ^3^Department of Biochemistry, Rajendra Institute of Medical Sciences, Ranchi, Jharkhand, India.

**Keywords:** Cell-free nucleic acid, Pregnancy, Messenger RNA, MicroRNA, Gestational diabetes.

## Abstract

**Background:**

Recent studies reveal an association between increased cell-free fetal (cff) nucleic acid in maternal blood and pregnancy challenges like loss, pre-eclampsia, growth restriction, and preterm labor.

**Objective:**

This article assesses the role of cff nucleic acids as potential diagnostic markers for the prediction and monitoring progression of severe pregnancy-related complications.

**Materials and Methods:**

In this systematic review and meta-analysis, various databases were searched. Original articles reporting on the role of cff nucleic acids in predicting the complications of pregnancy were included. I square test and funnel plot were used to analyze heterogeneity and publication bias, respectively. The quality of studies was assessed using the critical appraisal checklists for studies created by the Joanna Briggs Institute.

**Results:**

70 publications were selected for the final qualitative analysis. Articles were published between 2010 and 2023, and most studies were conducted in the USA and China. The majority of studies were conducted on the quantity of cff-DNA (n = 40), and the remaining on microRNA (n = 18), messenger RNA (n = 11), and cell-free RNA (n = 1). The pooled sensitivity of cff nucleic acids for detecting trisomy was found to be 90.9 (95% CI: 80.9–100%). MicroRNA levels were significantly increased in participants with gestational diabetes mellitus, with a standardized mean difference of 1.22 (95% CI: -0.90–3.34).

**Conclusion:**

Fetal nucleic acids can serve as accurate noninvasive diagnostic tools for predicting serious complications during pregnancy.

## 1. Introduction

In antenatal care, prenatal screening and diagnosis are routinely provided. It is believed that such amenities help women make decisions about whether to continue with pregnancies that are affected by developmental abnormalities. At present, the gold standard for prenatal diagnosis is the invasive method of acquiring fetal tissue and DNA. Though there is a little but considerable chance of miscarriage associated with invasive testing, many women prefer not to have it done (1, 2). To lower this risk of miscarriage and enable early testing, a reliable and feasible noninvasive prenatal diagnostic approach has long been sought upon.

The detection of cell-free fetal (cff) nucleic acids in the maternal bloodstream during pregnancy has completely changed the landscape of noninvasive prenatal screening and diagnosis. Circulating nucleic acids in maternal plasma and serum were anticipated to become a new area of interest in prenatal diagnostics following their discovery in 1997 (3). Although new in vivo studies show the possibility of other processes, cffDNA is released on trophoblastic cells as a result of apoptotic mechanisms. Since the rise in cffDNA levels occurs before the disease's clinical signs, it is highly valuable in the field of prenatal diagnosis and in predicting frequent pregnancy complications. Prenatal care has quickly included noninvasive prenatal screening utilizing cell-free DNA (cfDNA) in identifying common fetal aneuploidies, since its debut in 2011. According to a recent systematic review, the best sensitive and specific screening test for fetal Down syndrome, T13, and T18 in singleton and twin pregnancies is non-invasive prenatal screening with cfDNA. It was able to detect maternal diseases such as aneuploidies and malignancies, in contrast with conventional serum analyte screening (4).

While circulating cfDNA was the first known circulating nucleic acid, more have been discovered as molecular biology research and technology have advanced. At present, we take into consideration circulating nucleic acids in maternal plasma and serum, which is an array of extracellular nucleic acids that includes placental RNA, messenger RNA (mRNA), microRNA (miRNA), and other novel species like circular RNA, long noncoding RNA, mitochondrial DNA, and cfDNA (5). Alternative methods have been continuously explored, such as measuring total cfDNA or using gender-independent fetal epigenetic markers like DNA methylation and cfRNA of placental origin. These offer an appropriate replacement for genetic disease screening and prenatal diagnosis (6).

Recent research has demonstrated a correlation between elevated levels of cffDNA in maternal blood and various pregnancy complications, such as early pregnancy loss and preterm labor (7), pre-eclampsia (PE) (8–10), and fetal growth restriction (FGR) (11). Pregnancy difficulties have prompted studies to investigate the possibility of using cffDNA concentrations as a biomarker (12, 13). Research on the potential pathways connecting cffDNA to the etiology of pregnancy-related problems is only getting started (13).

We conducted a comprehensive review and meta-analysis to determine if the cff nucleic acid identification markers in the maternal bloodstream may be identified as diagnostic risk factors for fetal outcomes.

## 2. Materials and Methods

### Search strategy

A comprehensive literature search was conducted in 7 different databases (PubMed, The Cochrane Library, Embase, Science Direct, Web of Science, Scopus, and Google Scholar). Preferred reporting items for systematic review and meta-analyses (PRISMA) checklist guidelines were followed (14). Articles published between 2010 and October 2023 were included in the study. During the literature search, restrictions were not placed on country, time, or language of publication. Editorial letters, conference proceedings, and practice guidelines were excluded.

The following key terms were used to identify relevant studies: cffDNA, next-generation sequencing (NGS), ctDNA, fetal outcomes, fetal anomalies, cffDNA, cff nucleic acids, epigenetic methylation, noninvasive prenatal diagnosis, prenatal diagnosis, and genetic diagnosis. Only research articles were retrieved and reviewed. All possible combinations of keywords were utilized in the search. Table I shows the search strings used to retrieve research articles from each database. After removing duplicates, titles and abstracts were screened as per eligibility criteria. Full-text articles of all the identified abstracts were reviewed independently.

### Criteria for considering studies

Inclusion criteria encompass published research (case-control, cross-sectional, prospective, and retrospective cohorts) examining cff nucleic acid markers in maternal circulation. Studies on adult women ( ≥ 18 yr) with pregnancy-related complications were only included. Exclusion criteria encompass studies lacking relevance or adequate data, non-English publications, and certain study types (e.g., reviews, meta-analyses, letters to editors, commentaries, guidelines, book chapters, and editorials). Both published and unpublished studies, including gray literature, were considered for inclusion. The review also screened reference lists of prior systematic reviews/meta-analyses. Details such as authors, year of publication, country, study design, sample size, cell-free nucleic acid involved, pregnancy-related complications, and important results relevant to the study objectives were extracted from the included studies. The meta-analysis included studies based on specific criteria: relevant study design, availability of comparable outcome measures, sufficient statistical data, methodological quality, similar populations and interventions, and peer-reviewed full-text publications.

### Study quality assessment

The Joanna Briggs Institute's critical appraisal checklists for studies were used to assess the quality of selected studies (15). A study was deemed to have a high risk of bias if the “yes" score was 49% or lower, moderate risk if the score was between 50 and 69%, and low risk if the score was 70% or higher. Each study underwent bias assessment and classification as low risk, high risk, or with some concerns. Any discrepancies between independent reviewers were resolved by consulting a third reviewer.

### Statistical analysis and meta-analysis

For this meta-analysis, STATA version 17 was utilized. Continuous data were presented as means or medians with appropriate measures of variability. Categorical variables were expressed as numbers and percentages. A pooled meta-analysis was conducted. Heterogeneity was evaluated using Cochran's Q test and the DerSimonian and Laird method. Heterogeneity levels were categorized as low, moderate, or high based on I-square values. Low heterogeneity was defined as an I-square value of < 25%, moderate heterogeneity as 25–50%, and high heterogeneity as > 50%. Publication bias was assessed visually with funnel plots and quantitatively using Egger's test.

**Table 1:** Search strings used to retrieve articles from the databases

**Databases searched**	**Search strings used**
**PubMed (n = 1104)**	1. (“cell-free fetal nucleic acids") AND (“non-invasive prenatal diagnosis" OR “prenatal diagnosis" OR “genetic diagnosis") 2. (“cell-free fetal DNA" OR “NGS" OR “ctDNA" OR “cffDNA") AND (“non-invasive prenatal diagnosis" OR “prenatal diagnosis" OR “genetic diagnosis") 3. (“fetal outcomes" OR “fetal anomalies") AND (“non-invasive prenatal diagnosis" OR “prenatal diagnosis" OR “genetic diagnosis") 4. (“cell-free fetal nucleic acids") AND (“non-invasive prenatal diagnosis" OR “prenatal diagnosis" OR “genetic diagnosis") 5. (“epigenetic methylation") AND (“non-invasive prenatal diagnosis" OR “prenatal diagnosis" OR “genetic diagnosis")
**EMBASE (n = 1239)**	1. (`cell-free fetal nucleic acids'/exp OR “cell-free fetal nucleic acids") AND (`non-invasive prenatal diagnosis'/exp OR “non-invasive prenatal diagnosis" OR `prenatal diagnosis'/exp OR “prenatal diagnosis" OR `genetic diagnosis'/exp OR “genetic diagnosis") 2. (`cell-free fetal DNA'/exp OR “cell-free fetal DNA" OR `NGS'/exp OR “NGS" OR `ctDNA'/exp OR “ctDNA" OR `cffDNA'/exp OR “cffDNA") AND (`non-invasive prenatal diagnosis'/exp OR “non-invasive prenatal diagnosis" OR `prenatal diagnosis'/exp OR “prenatal diagnosis" OR `genetic diagnosis'/exp OR “genetic diagnosis") 3. (`fetal outcomes'/exp OR “fetal outcomes" OR `fetal anomalies'/exp OR “fetal anomalies") AND (`non-invasive prenatal diagnosis'/exp OR “non-invasive prenatal diagnosis" OR `prenatal diagnosis'/exp OR “prenatal diagnosis" OR `genetic diagnosis'/exp OR “genetic diagnosis") 4. (`cell-free fetal nucleic acids'/exp OR “cell-free fetal nucleic acids") AND (`non-invasive prenatal diagnosis'/exp OR “non-invasive prenatal diagnosis" OR `prenatal diagnosis'/exp OR “prenatal diagnosis" OR `genetic diagnosis'/exp OR “genetic diagnosis") 5. (`epigenetic methylation'/exp OR “epigenetic methylation") AND (`non-invasive prenatal diagnosis'/exp OR “non-invasive prenatal diagnosis" OR `prenatal diagnosis'/exp OR “prenatal diagnosis" OR `genetic diagnosis'/exp OR “genetic diagnosis")
**The Cochrane Library (n = 145)**	((“cell-free fetal nucleic acids" OR “cell-free fetal DNA" OR “NGS" OR “ctDNA" OR “cffDNA" OR “fetal outcomes" OR “fetal anomalies" OR “epigenetic methylation") AND (“non-invasive prenatal diagnosis" OR “prenatal diagnosis" OR “genetic diagnosis"))
**Google Scholar** **(n = 932)**	1.“cell-free fetal nucleic acids” AND “non-invasive prenatal diagnosis” OR “prenatal diagnosis” OR “genetic diagnosis” 2.“cell-free fetal DNA” OR “NGS” OR “ctDNA” OR “cffDNA” AND “non-invasive prenatal diagnosis” OR “prenatal diagnosis” OR “genetic diagnosis” 3.“fetal outcomes” OR “fetal anomalies” AND “non-invasive prenatal diagnosis” OR “prenatal diagnosis” OR “genetic diagnosis” 4.“cell-free fetal nucleic acids” AND “non-invasive prenatal diagnosis” OR “prenatal diagnosis” OR “genetic diagnosis” 5.“epigenetic methylation” AND “non-invasive prenatal diagnosis” OR “prenatal diagnosis” OR “genetic diagnosis”
**Science Direct** **(n = 1088)**	1. “cell-free fetal nucleic acids" AND (“non-invasive prenatal diagnosis" OR “prenatal diagnosis" OR “genetic diagnosis") 2. (“cell-free fetal DNA" OR “NGS" OR “ctDNA" OR “cffDNA") AND (“non-invasive prenatal diagnosis" OR “prenatal diagnosis" OR “genetic diagnosis") 3. (“fetal outcomes" OR “fetal anomalies") AND (“non-invasive prenatal diagnosis" OR “prenatal diagnosis" OR “genetic diagnosis") 4. “epigenetic methylation" AND (“non-invasive prenatal diagnosis" OR “prenatal diagnosis" OR “genetic diagnosis")
**Scopus (n = 766)**	1. TITLE-ABS-KEY(“cell-free fetal nucleic acids") AND TITLE-ABS-KEY(“non-invasive prenatal diagnosis" OR “prenatal diagnosis" OR “genetic diagnosis") 2. TITLE-ABS-KEY(“cell-free fetal DNA" OR “NGS" OR “ctDNA" OR “cffDNA") AND TITLE-ABS-KEY(“non-invasive prenatal diagnosis" OR “prenatal diagnosis" OR “genetic diagnosis") 3. TITLE-ABS-KEY(“fetal outcomes" OR “fetal anomalies") AND TITLE-ABS-KEY(“non-invasive prenatal diagnosis" OR “prenatal diagnosis" OR “genetic diagnosis") 4. TITLE-ABS-KEY(“cell-free fetal nucleic acids") AND TITLE-ABS-KEY(“non-invasive prenatal diagnosis" OR “prenatal diagnosis" OR “genetic diagnosis") 5. TITLE-ABS-KEY(“epigenetic methylation") AND TITLE-ABS-KEY(“non-invasive prenatal diagnosis" OR “prenatal diagnosis" OR “genetic diagnosis")
**Web of Science** **(n = 825)**	1. TS=(“cell-free fetal nucleic acids") AND TS=(“non-invasive prenatal diagnosis" OR “prenatal diagnosis" OR “genetic diagnosis") 2. TS=(“cell-free fetal DNA" OR “NGS" OR “ctDNA" OR “cffDNA") AND TS=(“non-invasive prenatal diagnosis" OR “prenatal diagnosis" OR “genetic diagnosis") 3. TS=(“fetal outcomes" OR “fetal anomalies") AND TS=(“non-invasive prenatal diagnosis" OR “prenatal diagnosis" OR “genetic diagnosis") 4. TS=(“cell-free fetal nucleic acids") AND TS=(“non-invasive prenatal diagnosis" OR “prenatal diagnosis" OR “genetic diagnosis") 5. TS=(“epigenetic methylation") AND TS=(“non-invasive prenatal diagnosis" OR “prenatal diagnosis" OR “genetic diagnosis")

## 3. Results

### Identification and description of studies

**Figure 1:**
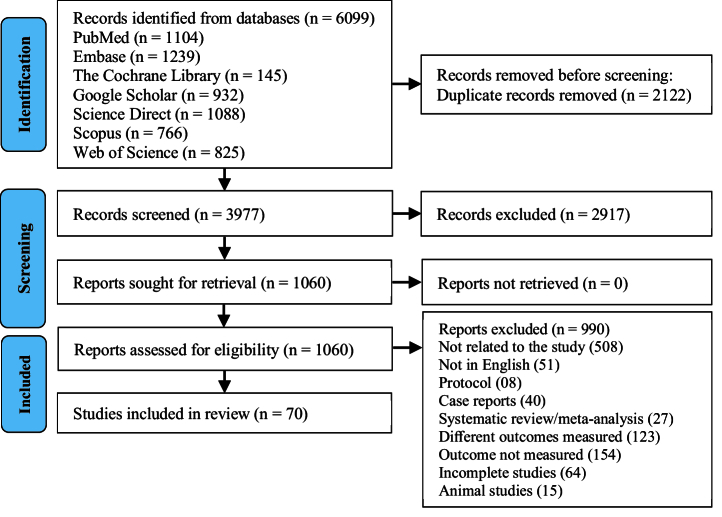
Flow chart depicting the process of selecting or rejecting studies. 70 studies were included in the systematic review. However, due to data heterogeneity, insufficient or incomplete statistical data, quality concerns, variations in outcome measures, and differences in study designs, 9 studies were included in the meta-analysis.

A total of 6099 citations were identified, with 2122 duplicate studies removed. These comprised 1104 from PubMed, 1239 from Embase, 145 from the Cochrane Library, 932 from Google Scholar, 766 from Scopus, 1088 from Science Direct, and 825 from Web of Science. Following the evaluation of titles and abstracts of 3977 articles, 2917 studies were excluded. Subsequently, 1060 articles underwent full-text review. After applying exclusion criteria, 990 complete texts were eliminated, leaving 70 articles for the final qualitative analysis. The study selection procedure is depicted in figure 1. This review, which includes 70 studies, aimed to determine whether the cff nucleic acid identifier markers in maternal circulation can be used as diagnostic risk factors for fetal outcomes. The publishing years were 2010–2023. Among 70 studies, 32 were prospective and retrospective cohorts, 29 were case-control, 08 were cross-sectional, and one study was randomized clinical trial. Most of the studies were conducted in the USA (n = 20), and among the remaining studies, 11 were from China, 7 from Italy, 4 from Australia, 3 each from the Czech Republic and Iran, 2 each from the UK, Denmark, Egypt, and Spain, 1 each from Belgium, Chile, Columbia, India, Israel, Japan, Malaysia, Mexico, Netherlands, Pakistan, Russia, South Africa, South Korea, and Turkey. The extracted data of the included studies are presented in table II.

### Cff nucleic acids and pregnancy complications

Among 70 studies, 8 studies included subjects with fetal aneuploidy, 12 studies with FGR, 11 studies with gestational diabetes mellitus (GDM), 6 studies with pregnancy-induced hypertension, 19 studies with preterm birth (PTB), 24 studies with PE, 4 studies with spontaneous abortion, and 6 studies with subjects having morbidly adherent placenta. The cff nucleic acids included were fetal DNA (n = 40), miRNA (n = 18), mRNA (n = 11), and cfRNA (n = 1).

**Figure 2:**
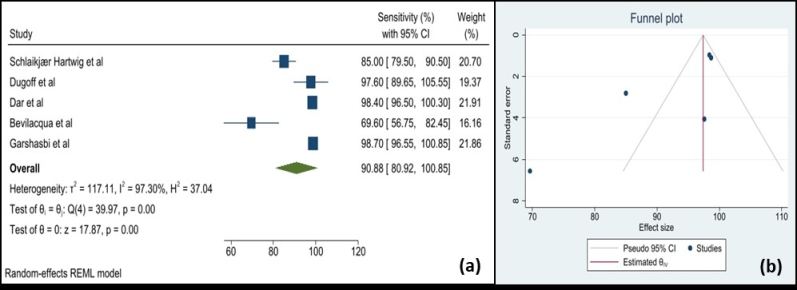
Forest and Funnel plots. A) Forest plot of sensitivity with 95% CI and weighted pooled summary statistics using a random-effects model in assessing cfDNA analysis in screening for fetal aneuploidy, B) Funnel plot of the publication bias.

**Figure 3:**
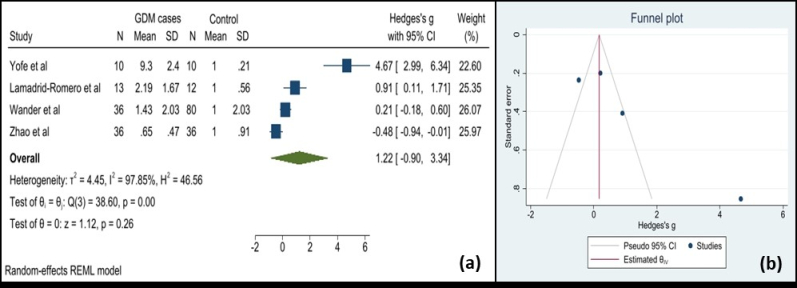
Forest and Funnel plot. A) Forest plot for studies using a random-effects model depicting the standardized mean difference of cfDNA in subjects with GDM vs. controls, B) Funnel plot of the publication bias.

Using maternal blood cfDNA analysis, trisomies 21, 18, and 13 may be reliably screened for. In our study, which adopted a random-effects model, the pooled sensitivity was 90.9 (95% CI: 80.9–100). In the meta-analysis (Figure 2), it was determined that the weighted pooled detection rate (DR) for the total number of 1963 cases of trisomy 21, 563 cases of trisomy 18, and 119 cases of trisomy 13 was 99.7% (95% CI: 99.1–99.9), 98.2% (95% CI: 95.5–99.2), and 99.0% (95% CI: 65.8–100), respectively. This led to a false-positive rate of about 0.1% overall among the over 200,000 pregnancies that were not impacted by these trisomies. This study revealed significantly increased miRNA levels in participants with GDM; the standardized mean difference was 1.22 (95% CI: -0.90–3.34) (Figure 3). However, the variability was high (I^2^ = 97.8%). There have been fewer studies exploring the relationship between cffDNA and GDM, but the results have been mixed. Another association between low cffDNA percentage and an elevated risk of GDM has been found in the first and second trimesters.

24 studies investigated first and second-trimester levels of cff nucleic acids; most of these studies showed a statistically significant increase in cfDNA concentrations in women who went on to develop PE. Following the analysis of circulating miRNA expression patterns in the 4 participants with PE, it was postulated that miRNA might be useful in both the diagnosis and prognosis of PE. 43 miRNAs had a pooled sensitivity of 0.86 (95% CI: 0.81–0.90), specificity of 0.89 (95% CI: 0.85–0.92), and an area under the curve (AUC) of 0.94 (95% CI: 0.91–0.96) in 15 trials with 2042 healthy controls and 2685 PE cases. It was also observed that preterm delivery (PTD)-affected women had higher levels compared to controls who were matched for gestational age and delivered infants at term. It was found that in both high and low-risk pregnancies, cffDNA testing in the first or second trimester eliminated any possible predictive value for premature birth. The incidence of PTD in a group at high risk due to early contractions or a history of spontaneous PTD was identified using a panel of 7 mRNAs that were analyzed between 24 and 28 gestational weeks. In the fetuses of individuals who gave birth to premature twins, certain miRNA signatures have been longitudinally detected throughout gestation. Regrettably, there is no way to verify the results because the miRNAs found and looked at in each investigation have differed.

Yet, only a small number of research have examined the connection and possible positive predictive value (PPV) of cffDNA levels for FGR. It is possible that FGR women's elevated DNase activity, relative to unaffected women, significantly affects their levels of cffDNA. Overall cfDNA values were observed to be higher in FGR cases throughout the third trimester. Few studies have looked closely at the amount of cffDNA in morbidly adherent placentas. The morbidly connected placenta in 7 instances in the third trimester had no aberrant cfDNA.

### Study quality assessment and publication bias

**Figure 4:**
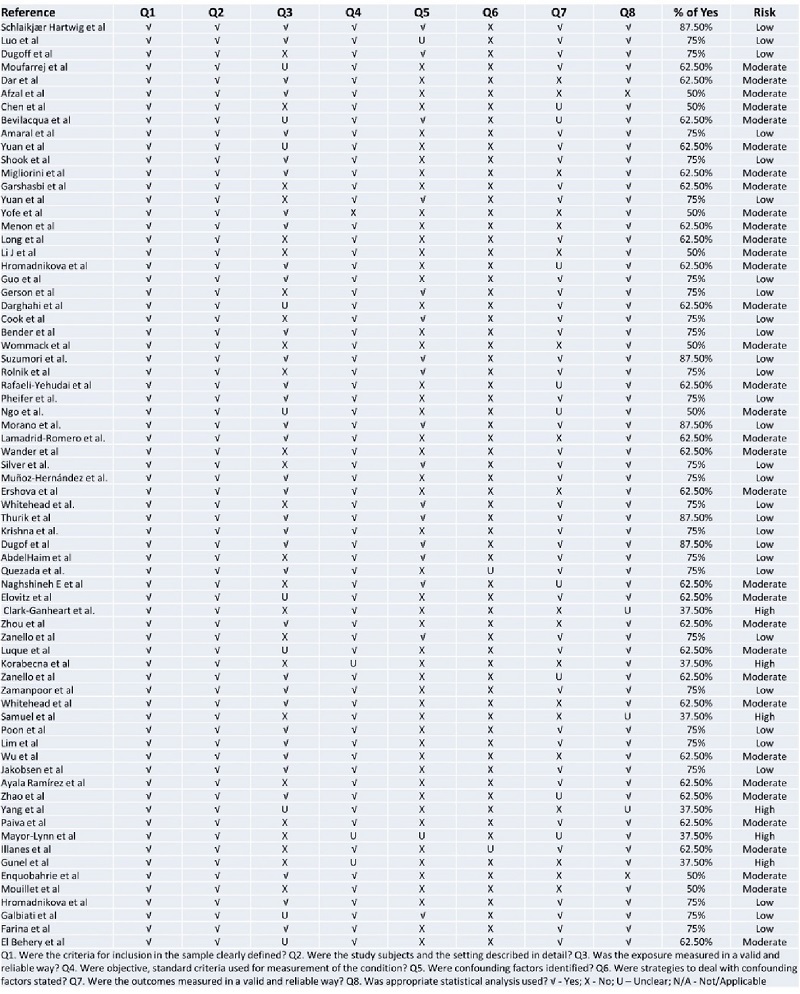
Quality assessment of studies.

2 independent reviewers assessed the quality of each included study. Most studies exhibited a low to moderate risk of bias, with a high percentage of positive responses to the Joanna Briggs Institute's tool questions. However, 6 studies were identified with a high risk of bias (Figure 4). Visual inspection of the funnel plots suggested asymmetry. Nevertheless, Egger's test indicated weak evidence for small-study effects (p > 0.05).

**Table 2:** Characteristics of included studies

**Author, yr (Ref)**	**Country**	**Study design**	**Sample size**	**cff nucleic acid involved**	**Pregnancy-related complication**	**Results**	**Risk of bias**
**Schlaikjær Hartwig ** ***et al*** **., 2023 (16)**	Denmark	Cohort	n = 333 with a pregnancy loss n = 667 to evaluate cffDNA performance	Fetal DNA	Fetal aneuploidy	Of 1000 cffDNA tests, 11% were inconclusive, 45% euploid, 41% aneuploid, and 4% had multiple aneuploidies. cffDNA may differentiate pregnancy loss types	Low
**Luo ** ***et al*** **., 2023 (7)**	China	Cohort	n = 13,292 cases in the term group n = 728 cases in the spontaneous preterm group	Fetal DNA	PTB	Maternal age and BMI inversely correlate with fetal cfDNA proportion. No link exists between fetal fraction on NIPT and later spontaneous preterm delivery	Low
**Dugoff ** ***et al*** **., 2023 (17)**	USA	Cohort	n = 1447 cases	Fetal DNA	Fetal aneuploidy	Trisomy 21 was detected in 41 of 42 pregnancies (97.6% rate). cfDNA testing is effective for trisomy 21 screening in twin gestations from the 1^st^ trimester	Low
**Moufarrej ** ***et al*** **., 2022 (8)**	USA	Cohort	n = 199 pregnant mothers	cfRNA	PE	Early in gestation, *cfRNA* gene expression differs significantly between normotensive and preeclamptic mothers	Moderate
**Dar ** ***et al*** **., 2022 (18)**	USA	Cohort	(13,043 low-risk and 4808 high-risk cases for aneuploidy) n = 100 trisomy 21 n = 18 trisomy 18 n = 15 trisomy 13	Fetal DNA	Fetal aneuploidy	SNP-based cfDNA shows high specificity and sensitivity in low-risk women, with an 85.7% PPV for trisomy 21	Moderate
**Afzal ** ***et al*** **., 2022 (19)**	Pakistan	Cohort	n = 26 with beta-thalassemia (13 with homozygous mutation and 13 with heterozygous mutations)	Fetal DNA	Beta-thalassemia	Maternal cfDNA in NIPT may detect paternally inherited mutant alleles, potentially avoiding invasive treatments	Moderate
**Chen ** ***et al*** **., 2021 (20)**	China	Cohort	n = 5 dichorionic diamniotic twins	Fetal DNA	Fetal aneuploidy	False-positive NIPT results may occur up to 16 wk after cotwin death due to residual cfDNA following selective reduction at 14–17 wk	Moderate
**Bevilacqua ** ***et al*** **., 2021 (21)**	Belgium	Cohort	n = 358 prospectively enrolled pregnancies n = 377 stored clinical samples	Fetal DNA	Fetal aneuploidy	Nested 22q11.2 deletions are common but minor, with cfDNA tests showing a low false-positive rate (0-0.5%)	Moderate
**Amaral ** ***et al*** **., 2021 (22)**	USA	Cohort	n = 98 healthy pregnancies n = 88 gestational hypertension n = 91 with PE	Fetal DNA	HDP	Gestational hypertension and PE show higher total cfDNA levels (197.0 and 174.2 ng/mL) than healthy pregnancies (140.5 ng/mL; both p < 0.0001)	Low
**Yuan ** ***et al*** **., 2020 (23)**	China	Cohort	n = 523 GDM n = 88 ICP n = 64 PE n = 62 PIH n = 150 PTB	Fetal DNA	GDM, ICP, PE, PIH and PTB	Low fetal fraction increases risk of early PTB ( < 34 wk: OR = 3.09) and PE (OR = 2.06)	Moderate
**Shook ** ***et al*** **., 2020 (24)**	USA	Cohort	n = 2033 women	Fetal DNA	Fetal aneuploidy	Infants < 5 ${ߐ}^{\text{th}}$ percentile are more likely born to women with high FF in the 1^st^ trimester. High FF does not significantly correlate with premature delivery or pregnancy-related hypertension	Low
**Migliorini ** ***et al*** **., 2020 (25)**	Italy	RCT	n = 40 women	Fetal DNA	Fetal aneuploidy	Women in the cfDNA group had higher satisfaction, reassurance, and lower anxiety scores	Moderate
**Garshasbi ** ***et al*** **., 2020 (6)**	Iran	Cohort	n = 94 T21, n = 39 T18, n = 8 T13, n = 15 XO, n = 6 XXX, n = 3 XYY, n = 5 XXY and n = 11,042 euploid	Fetal DNA	Fetal aneuploidy	In mixed-risk pregnancies, NIPT excelled in screening for fetal T21, T18, T13, and SCA	Moderate
**Yuan ** ***et al*** **., 2019 (26)**	China	Cohort	n = 111 GDM n = 39 ICP n = 52 PE n = 630 controls	Fetal DNA	PE ICP GDM	Elevated cfDNA levels in the second trimester were significantly linked to higher risks of PE, ICP, and GDM compared to controls	Low
**Yoffe ** ***et al*** **., 2019 (27)**	Italy	Case-control	n = 23 GDM n = 20 controls	miRNA	GDM	MiR-223 and miR-23a may indicate GDM in the first trimester. MiR-223 alone has the strongest predictive value with an AUC of 0.94	Moderate
**Menon ** ***et al*** **., 2019 (28)**	India	Case-control	n = 10 preterm n = 20 controls	miRNA	PTB	Distinct miRNA patterns were found in women who gave birth at term vs. those who delivered prematurely	Moderate
**Long ** ***et al*** **., 2019 (29)**	China	Case-control	n = 23 placenta accrete n = 27 controls	mRNA	Placenta accreta	VEGF mRNA, osteopontin, and miR-518b were higher in placental tissue of women with placenta accreta	Moderate
**Li ** ***et al*** **., 2019 (30)**	China	Case-control	n = 21 placenta previa n = 12 placenta previa and accrete n = 35 controls	mRNA	Placenta accreta	High hPL mRNA levels are higher than in controls but cannot predict if an invasive placenta will lead to hysterectomy	Moderate
**Hromadnikova ** ***et al*** **., 2019 (31)**	Czech Republic	Case-control	n = 43 PE n = 63 FGR n = 102 controls	miRNA	PE FGR	Women with gestational hypertension have downregulated miR-517-5p, miR-520a-5p, and miR-525-5p. Women who develop FGR show downregulation of miR-520a-5p	Moderate
**Guo ** ***et al*** **., 2019 (32)**	China	Cohort	n = 55 early PTB ( < 34 wk) n = 284 late preterm (34-37 wk) n = 8129 controls	Fetal DNA	PTB	No correlation was found between fetal fraction DNA in the second trimester and early or late preterm delivery	Low
**Gerson ** ***et al*** **., 2019 (33)**	USA	Cohort	n = 639 pregnancies undergoing cfDNA testing	Fetal DNA	PTB PE PIH	Low FF was linked to placental compromise PIH (RR 1.6), and severe PE (RR 3.3). Preterm delivery was not linked to low FF	Low
**Darghahi ** ***et al*** **., 2019 (34)**	Iran	Case-control	n = 50 PTB n = 50 controls	Fetal DNA	PTB	Higher free DNA levels are found in women who give birth prematurely	Moderate
**Cook ** ***et al*** **., 2019 (35)**	UK	Cohort	n = 50 preterm with a short cervix	miRNA	PTB	MiRNA levels can predict cervical shortening (AUC = 0.85) and premature birth (AUC = 0.87)	Low
**Bender ** ***et al*** **., 2019 (36)**	USA	Cohort	n = 221 PIH or PE n = 166 severe PE n = 2314 controls	Fetal DNA	PE PIH	First-trimester fetal DNA levels were significantly lower in women with hypertension disorders compared to controls	Low
**Wommack** *** et al*** **., 2018 (37)**	USA	Cross-sectional	n = 21 PTB n = 21 controls	miRNA	PTB	Certain miRNA clusters were linked to gestational duration	Moderate
**Suzumori ** ***et al*** **., 2018 (38)**	Japan	Case-control	n = 131 PIH n = 100 GDM n = 44 preterm n = 37 spontaneous abortions n = 5270 controls	Fetal DNA	PIH GDM PTB Spontaneous abortion	PIH correlated with low fetal DNA proportions, but no correlation was found when accounting for other pregnancy issues	Low
**Rolnik ** ***et al*** **., 2018 (39)**	Australia	Cohort	n = 4317 pregnant women	Fetal DNA	PE FGR	Decreased first-trimester fetal DNA percentage significantly correlates with increased risk of FGR and PE	Low
**Rafaeli-Yehudai ** ***et al*** **., 2018 (11)**	Israel	Cross-sectional	n = 21 PE n = 28 FGR n = 39 controls	Fetal DNA	PE FGR	Maternal serum cfDNA levels in PE are greater than in normal pregnancy or FGR	Moderate
**Pheiffer ** ***et al*** **., 2018 (40)**	South Africa	Case-control	n = 28 GDM n = 53 controls	miRNA	GDM	MiR-20a-5p, combined with other risk factors, may accurately predict GDM onset	Low
**Ngo ** ***et al*** **., 2018 (41)**	USA	Cohort	n = 13 preterm n = 25 controls	mRNA	PTB	Some mRNA sequences might predict PTD up to 2 months in advance	Moderate
**Morano ** ***et al*** **., 2018 (42)**	Italy	Case-control	n = 231 pregnant women	Fetal DNA	FGR	The expected cfDNA fetal fraction is smaller in FGR with an early start ( < 32 wk)	Low
**Lamadrid-Romero ** ***et al*** **., 2018 (43)**	Mexico	Cohort	n = 67 GDM n = 74 controls	miRNA	GDM	Higher miR-125b-5p levels were found in pregnancies leading to GDM	Moderate
**Wander ** ***et al*** **., 2017 (44)**	USA	Case-control	n = 35 GDM n= 80 controls	miRNA	GDM	MiR-155-5p and miR-2-3p, examined in early to mid-pregnancy, were significantly linked to GDM risk	Moderate
**Silver ** ***et al*** **., 2017 (45)**	USA	Case-control	n = 175 PE n = 175 controls	Fetal DNA	PE	Women who developed PE in the first trimester had no significant difference in total cfDNA levels	Low
**Mu noz-Hernández ** ***et al*** **., 2017 (46)**	Spain	Cohort	n = 37 mild PE n = 25 severe PE n = 16 HELLP syndrome n = 26 controls	Fetal DNA	PE HELLP	Total cfDNA marker concentrations were higher in pre-eclamptic and HELLP women compared to controls	Low
**Ershova ** ***et al*** **., 2017 (47)**	Russia	Cohort	n = 40 FGR n = 40 controls with normal pregnancy n = 40 controls healthy nonpregnant women	Fetal DNA	FGR	Activating the cfDNA elimination mechanism could decrease cfDNA levels in blood	Moderate
**Whitehead ** ***et al*** **., 2016 (48)**	Australia	Case-control	n = 40 late onset FGR n = 80 controls	mRNA	FGR	Certain mRNA profiles distinguish women with late-onset FGR from those with normal gestation	Low
**Thurik ** ***et al*** **., 2016 (49)**	Netherlands	Case-control	n = 37 PE n = 84 PIH n = 56 GDM n = 49 PTB n = 301 controls	Fetal DNA	PE PTB GDM PIH	Fetal DNA was significantly linked to either GDM or PIH. Fetal DNA did not correlate with premature birth or PE	Low
**Krishna ** ***et al*** **., 2016 (50)**	USA	Cohort	n = 370 n = 22 low fetal fractions n = 345 controls (sufficient fetal fraction)	Fetal DNA	Spontaneous abortion Stillbirth PTB GDM pPROM FGR	Women with pPROM, GDM, and premature delivery had lower fetal DNA fractions. No correlation was found between spontaneous abortion and IUGR	Low
**Dugoff ** ***et al*** **., 2016 (51)**	USA	Cohort	n = 119 PTB ( < 37 wk) n = 49 very PTB ( < 34 wk) n = 1181 control	Fetal DNA	PTB	Elevated cfDNA fetal percentage between 14 and 20 wk was significantly linked to more common PTB and extreme PTB	Low
**AbdelHaim ** ***et al*** **., 2016 (52)**	Egypt	Cohort	n = 51 mild PE n = 56 severe PE n = 93 controls	Fetal DNA	PE + SGA	cfDNA levels in severe PE were over 6 times higher than in controls and about twice as high as in mild PE	Low
**Quezada ** ***et al*** **., 2015 (53)**	UK	Cohort	n = 103 PTB n = 3066 control	Fetal DNA	PTB	Fetal fraction measured at 11-13 wk gestation does not indicate postnatal death	Low
**Naghshineh ** ***et al*** **., 2015 (54)**	Iran	Cross-sectional	n = 12 placenta accrete n = 38 controls	miRNA	Placenta accreta	With an AUC of 0.94, mRNA strongly predicts placenta accreta, with or without complications	Moderate
**Elovitz ** ***et al*** **., 2015 (55)**	USA	Case-control	n = 40 PTB n = 40 controls	miRNA	PTB	The risk of premature delivery was not correlated with miRNA levels	Moderate
**Clark-Ganheart ** ***et al*** **., 2015 (56)**	USA	Cohort	n = 50 miscarriage	Fetal DNA	Spontaneous abortion	After 8 wk' gestation, cfDNA with fetal fractions > 3.7% is found in maternal plasma of nonviable pregnancies in over 75% of cases	High
**Zhou ** ***et al*** **., 2014 (57)**	China	Case-control	n = 21 placenta previa n = 12 placenta previa/accrete n = 35 controls	mRNA	Placenta previa/accreta	Women with placenta accreta, especially those who had a hysterectomy, had significantly higher cell-free β-HCG mRNA levels	Moderate
**Zanello ** ***et al*** **., 2014 (58)**	Italy	Case-control	n = 43 PE n = 200 controls	mRNA	PE	Combining PLAC1 with indicators like mean arterial pressure and PE history may enable early PE diagnosis	Low
**Luque ** ***et al*** **., 2014 (59)**	Spain	Case-control	n = 31 PE n = 44 controls	miRNA	PE	Maternal miRNA levels in the first trimester were not correlated with the likelihood of developing PE	Moderate
**Korabecna ** ***et al*** **., 2014 (60)**	Czech Republic	Case-control	n = 4 APS n = 21 controls	Fetal DNA	APS	No statistically significant difference was found between participants' and controls' cfDNA levels	High
**Zanello ** ***et al*** **., 2013 (61)**	Italy	Case-control	n = 46 FGR n = 42 controls	mRNA	FGR	In women impacted by FGR, there was an increase in maternal mRNA EGFL7	Moderate
**Zamanpoor ** ***et al*** **., 2013 (62)**	Malaysia	Case-control	n = 40 GDM n = 35 PIH n = 38 controls	Fetal DNA	GDM PIH	GDM does not affect fetal DNA in maternal plasma, but PIH significantly increases cffDNA levels in the third trimester	Low
**Whitehead ** ***et al*** **., 2013 (63)**	Australia	Cohort	n = 20 FGR n = 15 PTB n = 8 controls	mRNA	FGR	mRNA expression variations in the mother blood of women with severe preterm FGR	Moderate
**Samuel ** ***et al*** **., 2013 (64)**	USA	Case-control	n = 7 placenta accrete n = 6 placenta previa n = 7 controls	Fetal DNA	Placenta previa/accreta	The average percentage of cfDNA does not vary much between the groups	High
**Poon ** ***et al*** **., 2013 (65)**	USA	Cohort	n = 46 PE n = 20 PTB < 34 wk n = 68 FGR n = 1805 controls	Fetal DNA	PE PTB SGA	No apparent change in fetal DNA is observed at 11–13 wk in pregnancies with SGA, premature delivery, or PE	Low
**Lim ** ***et al*** **., 2013 (66)**	South Korea	Case-control	n = 41 abortions with normal karyotype n = 26 abortions with abnormal karyotype n = 220 controls	Fetal DNA	Spontaneous abortion	Spontaneous abortion-affected pregnancies had higher levels of fetal-free DNA, regardless of karyotype abnormalities	Low
**Wu ** ***et al*** **., 2012 (67)**	China	Case-control	n = 10 PE n = 9 controls	miRNA	PE	Pre-eclamptic women had higher levels of miR-24, miR-26a, miR-103, miR-130b, miR-181a, miR-342-3p, and miR-574-5p than controls	Moderate
**Jakobsen ** ***et al*** **., 2012 (68)**	Denmark	Cohort	n = 847 fetal-free DNA ≤ 95^th^ percentile n = 43 fetal-free DNA > 95^th^ percentile n = 19 PTB ( < 37 wk) n = 8 very PTB ( < 34 wk)	Fetal DNA	PTB	Women with fetal-free DNA above the 95^th^ percentile had a higher risk of PTD. ORs for PTB before 37 wk and 34 wk are 6.3 and 16.6, respectively	Low
**Ayala Ramírez ** ***et al*** **., 2012 (69)**	Colombia	Case-control	n = 8 FGR n = 21 controls	Fetal DNA	FGR	No statistically significant variations were found in *hPL* gene total RNA or mRNA expression between groups	Moderate
**Zhao ** ***et al*** **., 2011 (70)**	China	Case-control	n = 24 GDM n = 24 controls	miRNA	GDM	MiRNA levels differed between normal pregnancies and GDM. Some miRNA sequences may become potential biomarkers for early GDM detection	Moderate
**Yang ** ***et al*** **., 2011 (71)**	China	Cross-sectional	n = 4 PE n = 1 controls	miRNA	PE	The expression of miRNA is elevated in pre-eclamptic women	High
**Paiva ** ***et al*** **., 2011 (72)**	Australia	Cross-sectional	n = 21 PE n = 15 controls	mRNA	PE	Preeclamptic women had considerably greater levels of PLAC3, PLAC4, CRH, and syncytin than controls	Moderate
**Mayor-Lynn ** ***et al*** **., 2011 (73)**	USA	Cross-sectional	n = 7 PE n = 7 preterm n = 7 controls	miRNA	PE PTB	Women with PTD or PE had significantly different placental miRNA expression compared to controls with normal gestational age	High
**Illanes ** ***et al*** **., 2011 (74)**	Chile	Case-control	n = 20 cervix < 15 mm PTD n = 20 cervix < 15 mm delivery at term n = 22 normal cervix	Fetal DNA	PTB	No apparent relationship was found between PTD and fetal DNA, and no differences in fetal DNA were observed among the 3 groups	Moderate
**Gunel ** ***et al*** **., 2011 (75)**	Turkey	Case-control	n = 20 PE n = 20 controls	miRNA	PE	In pregnant women at risk of PE, miR-210 levels are more than 2 times greater than miR-153 levels	High
**Enquobahrie ** ***et al*** **., 2011 (76)**	USA	Cross-sectional	n = 20 PE n = 20 controls	miRNA	PE	In women with PE, miR-210 was significantly elevated, while miR-328, miR-584, miR-139-5p, miR-500, miR-1247, miR-34c-5p, and miR-1 were downregulated in placental expression	Moderate
**Mouillet ** ***et al*** **., 2010 (77)**	USA	Cross-sectional	n = 14 FGR n = 14 controls	miRNA	FGR	Women with FGR had higher miRNA levels compared to those with normal pregnancies	Moderate
**Hromadnikova ** ***et al*** **., 2010 (78)**	Czech Republic	Case-control	n = 24 PE n = 11 FGR n = 18 risk of placental insufficiency n = 70 controls	Fetal DNA	PE FGR	Participants with FGR and PE had considerably higher plasma concentrations of fetal DNA	Low
**Galbiati ** ***et al*** **., 2010 (79)**	Italy	Case-control	n = 12 PE n = 10 FGR n = 12 controls	Fetal DNA	PE SGA	Women with overt PE had significantly higher levels of fetal DNA and CRH mRNA. Women developing SGA had only higher fetal DNA levels	Low
**Farina ** ***et al*** **., 2010 (80)**	Italy	Cohort	n = 11 PE n = 88 controls	mRNA	PE	Pre-eclamptic women had higher endoglin, FLT1, and TGFB1 mRNA levels, and lower PlGF and PP13 mRNA levels	Low
**El Behery ** ***et al*** **., 2010 (81)**	Egypt	Cohort	n = 7 placenta accrete n = 28 controls	mRNA	Placental previa/accreta	Women with placenta accreta showed higher levels of mRNA hPL	Moderate
Cf: Cell-free, cffDNA: Cell-free fetal DNA, PTB: Preterm birth, BMI: Body mass index, NIPT: Noninvasive prenatal testing, cfDNA: Cell-free DNA, cfRNA: Cell-free RNA, PE: Pre-eclampsia, FPR: False positive rate, GDM: Gestational diabetes mellitus, ICP: Intrahepatic cholestasis of pregnancy, PIH: Pregnancy-induced hypertension, FF: Fetal fraction, SCA: Sickle cell anemia, AUC: Area under the curve, miRNA: MicroRNA, mRNA: Messenger RNA, VEGF: Vascular endothelial growth factor, hPL: Human placental lactogen, FGR: Fetal growth restriction, RR: Relative risk, HELLP: Hemolysis, elevated liver enzymes, and low platelet count, PROM: Preterm premature rupture of membranes, SGA: Small for gestational age, β -HCG: Beta-human chorionic gonadotropin, PLAC: Placental growth factor, APS: Antiphospholipid syndrome, EGFL7: Epidermal growth factor-like 7, PTD: Preterm delivery, CRH: Corticotropin-releasing hormone, FLT1: Fms-related tyrosine kinase 1, TGFB1: Transforming growth factor beta 1, PlGF: Placental growth factor, PP13: Pregnancy-associated plasma protein A							

## 4. Discussion

Prenatal diagnostics and complicated pregnancies are 2 areas of obstetrical study that have promoted the use of cffDNA, a novel and promising biomarker. It is commonly known that circulating amounts of cffDNA contribute to several complications during pregnancy. While intriguing in vivo studies show the possibility of other processes, cffDNA is produced on trophoblastic cells as a result of apoptotic mechanisms. Because it occurs before the illness exhibits clinical signs, the rise in cffDNA levels is highly valuable for prenatal diagnosis as well as for predicting frequent complications associated with pregnancy. The available research was compiled in this systematic review to determine whether circulating nucleic acids in mother plasma and serum could function as additional, independent markers for the prediction and/or monitoring of the most common, severe pregnancy complications.

Reliable trisomies 21, 18, and 13 screening is provided by maternal blood cfDNA analysis. In the meta-analysis, it was determined that the weighted pooled DR for the total number of 1963 cases of trisomy 21, 563 cases of trisomy 18, and 119 cases of trisomy 13 was 99.7%, 98.2%, and 99.0%, respectively. The false-positive rate appeared to be equally low, although the reported DR of trisomy 21 by cfDNA testing was high but less than that in singleton pregnancies, as noted in an updated meta-analysis in twin pregnancies (82). Trisomy 21 was detected in 41 out of 42 pregnancies, reaching a 97.6% DR (17). The distinction between euploid and aneuploid pregnancy loss may be possible using cffDNA-based diagnostics, according to recent research (16).

During pregnancy, GDM is a temporary abnormal glucose tolerance that is identified in the latter half or third trimester of the pregnancy. Complications from GDM are quite likely to affect both the mother and the offspring. It has been proposed that circulating RNAs, particularly miRNAs, may be potential biomarkers for the early identification of GDM. MiRNA levels in participants with GDM were found to be considerably higher in this investigation; the standardized mean difference was 1.22 (95% CI: -0.90–3.34). According to a recent meta-analysis, circulating levels of miR-132 and miR-155 were lowered in participants with GDM, whereas miR-29a, miR-330, miR-134, miR-16, miR-223, and miR-17 showed substantial overall increase in GDM (83). With an odds ratio of 3.24, reduced expression of miR-20a-5p in females with one or more biophysical risk factors was found to be a strong predictor of GDM (40). Another study in 41 individuals during the first trimester discovered that pregnancies that would develop GDM had approximately 1.7-fold higher values for miR-125b-5p compared to the control group (43). A few studies have explored the relationship between cffDNA and GDM, but the findings have been inconsistent. While one study observed that cffDNA measured in the first trimester was significantly lower in women who later developed GDM (49), another found no difference in the second or third trimester (62). Additionally, low cffDNA percentages during the first and second trimesters have been linked to increased GDM risk in other studies (50, 26). Conversely, elevated levels were observed in women over 35 affected by GDM (23).

A pregnancy condition known as PE is characterized by an onset of proteinuria and hypertension after 20 wk of gestation. According to recent studies, genetic indicators could influence the likelihood of PE (67). In this study, a statistically significant rise in cfDNA concentrations were found in women who went on to develop PE. However, one study concluded that cfDNA is not a useful marker for PE after analyzing a large dataset of over 5000 cases (38). In contrast, after evaluating the circulating miRNA expression patterns in 4 participants with PE, it was hypothesized that miRNA could be beneficial for both the diagnosis and prognosis of PE (71). Another study found significant and consistent differences in *cfRNA* gene expression between normotensive and pre-eclamptic mothers early in gestation, well before symptoms appear (8). In 15 studies involving 2042 healthy controls and 2685 participants with PE, 43 miRNAs showed a pooled sensitivity of 0.86 (95% CI: 0.81–0.90), specificity of 0.89 (95% CI: 0.85–0.92), and an AUC of 0.94 (95% CI: 0.91–0.96). Another analysis from 8 papers, including 343 normal pregnancies and 273 participants with PE, revealed a diagnostic odds ratio of 50.24 (95% CI: 21.28–118.62), specificity of 0.87 (95% CI: 0.78–0.92), and pooled sensitivity of 0.88 (95% CI: 0.80–0.93). They concluded that there is a reasonably good diagnostic and predictive accuracy in using circulating miRNAs to differentiate participants with PE from controls (10).

An early delivery, occurring before 37 wk of gestation, is referred to as PTD. The majority of PTDs are spontaneous, resulting from preterm labor or preterm premature rupture of membranes. It has been proposed that maternal plasma cffDNA could serve as a marker for preterm labor. One study found that increased cffDNA levels at 14–20 wk were associated with a higher risk of PTD at both < 34 wk and < 37 wk (51); however, other studies did not identify any connection between cffDNA and PTD during the second trimester (32, 53). Similarly, another study found that PTD at < 37 wk was linked to cfDNA levels in the second trimester (50). Additionally, a recent study in China reported an inverse correlation between body mass index, maternal age, and the cell-free proportion of fetal DNA.

A panel of 7 mRNAs analyzed at 24–28 gestational weeks was found to detect the incidence of PTD in a high-risk group with early contractions or a history of spontaneous PTD (41). Certain miRNA signatures have been longitudinally identified during gestation in the fetuses of those who experienced a PTD birth. Unfortunately, the miRNAs identified and examined in each study have varied, and there is no way to validate the findings.

FGR is a diverse clinical syndrome linked to a higher risk of perinatal morbidity and death as well as fetal deterioration. The primary source of the increased cffDNA seen in participants with symptomatic FGR has been identified as placental malfunction, which is characteristic of FGR. Although limited studies have looked at the relationship and potential PPV of cffDNA levels for FGR. DNase activity, which is higher in FGR women than in unaffected women, may have a major impact on cffDNA levels in these women, according to a recent cohort study (47). The relationship between FGR and total DNA was examined in very few studies. In FGR instances, Mu noz-Hernández et al. (46) discovered greater total cfDNA readings throughout the third trimester. A single observation found that increased cfDNA was associated with FGR (24), but another study did not find a predictive effect of total cfDNA for FGR in the first trimester (65). Additionally, a recent study indicated that circulating miRNAs assessed were not accurate indicators for FGR prediction during the first trimester (31).

Morbidly adherent placentas are a broad range of placental defects caused by abnormal trophoblast invasion into the uterine wall's myometrium. Although a number of sonographic characteristics associated with invasive placentation have been identified, they lack sensitivity and specificity. As a result, additional markers may be helpful for monitoring the disease's course and facilitating early identification. The quantity of cffDNA in morbidly adherent placenta has not been thoroughly studied. In a study involving 7 cases in the third trimester, no abnormal cfDNA was found in the morbidly adherent placenta (64). However, other studies reported that human placental lactogen mRNA levels were 3 times higher (81, 30), and higher levels of miR-518b were observed in a case-control study (29). More research is needed to determine whether miRNA plays a role in predicting and monitoring morbidly adherent placentas.

## 5. Conclusion

Maternal care would be greatly influenced by a noninvasive, sensitive test that might detect pregnant women who are at high risk of developing complications early in the pregnancy. In addition to increasing clinical monitoring and perhaps lowering maternal-fetal morbidity as well as mortality, it would present a chance to implement preventive treatments for a subset of participants. Cff nucleic acids, if looked at in the first trimester, may prove to be a reliable noninvasive diagnostic tool for predicting serious complications during pregnancy. To validate this relationship, additional research is necessary.

##  Data Availability

Data supporting the findings of this study are available upon reasonable request from the corresponding author.

##  Author Contributions

JR. Keshari and P. Prakash designed the study and conducted the research. JR. Keshari, P. Prakash, SR. Sinha, P. Prakash, K. Rani, T. Aziz, and Sh. Shilpa monitored, evaluated, and analyzed the study results. Further, JR. Keshari, P. Prakash, SR. Sinha, P. Prakash, K. Rani, T. Aziz, and Sh. Shilpa reviewed the article. All authors approved the final manuscript and take responsibility for the integrity of the data.

##  Conflict of Interest

The authors declare that there is no conflict of interest.
